# A review on clinical implications of S100 proteins in lung diseases

**DOI:** 10.3389/fmed.2025.1618772

**Published:** 2025-10-14

**Authors:** Vineesh V. Raveendran, Somaya AlQattan, Eid AlMutairy

**Affiliations:** Lung Health Centre, King Faisal Specialist Hospital and Research Centre, Riyadh, Saudi Arabia

**Keywords:** S100A proteins, lung diseases, pulmonary fibrosis, lung transplantation, COVID-19

## Abstract

The S100 family of proteins plays a pivotal role in the pathogenesis of lung diseases, including asthma, chronic obstructive pulmonary disease (COPD), cystic fibrosis, pulmonary arterial hypertension (PAH), pulmonary fibrosis, lung cancers, acute lung injury, acute respiratory distress syndrome, COVID-19, and lung transplantation. This review comprehensively examines the contributions of S100 proteins to the progression of these disorders, focusing on their potential as diagnostic and prognostic biomarkers, as well as therapeutic targets. S100A protein-mediated key molecular mechanisms that influence inflammation, airway remodeling, fibrosis, and tumorigenesis in the lungs are discussed. The importance of their normal function is evident from the observation that simultaneous mutations in S100A3 and S100A13 predispose individuals to early-onset pulmonary fibrosis, underscoring their critical role in lung health. Furthermore, sustained S100 protein elevation is explored in the context of long COVID, shedding light on its role in chronic inflammation. These proteins act as damage-associated molecular patterns (DAMPs), activating immune pathways via receptors like TLR4 and RAGE, thereby driving inflammation and immune cell recruitment. Notably, in lung transplantation, elevated levels of S100A8, S100A9, and S100A12 serve as early biomarkers of graft rejection and complications such as graft-vs.-host disease, which indicates their role in mediating immune responses and transplant outcomes. While promising, the clinical application of S100 proteins faces challenges, including disease-specific variability and the need for robust validation across diverse populations. This narrative review underscores the dual potential of S100 proteins as biomarkers and therapeutic targets in respiratory medicine while emphasizing the importance of overcoming current limitations through targeted research and clinical trials.

## Introduction

Human diseases have long been associated with the dysregulation of protein expression and functions, which play pivotal roles in maintaining cellular homeostasis (1, 2). Proteins are the driving force of signal transduction, structural maintenance, enzymatic catalysis, and immunological responses, and the perturbations in their expression levels or functional integrity because of genetic alterations, environmental influences, or other factors can result in diseases (1, 3, 4). Many protein families, like S100 family, are evolutionarily conserved to carry out the fundamental processes that maintain the physiological homeostasis of an organism (5, 6). S100 protein family, (Table 1) (7–9) comprises S100A1 to S100A16, S100B, S100G, S100P, and S100Z (10), along with S100-fused-type proteins such as trichohyalin (11), filaggrin (12), filaggrin2 (13), cornulin (14), and repetin (15) (*see* Table 1 *for general details. We are not including S100-fused-type proteins in the table, as the relation of these proteins in lung diseases is almost null)*.

**Table 1:** General characteristics of S100 proteins.

**S100 protein**	**Salient features**	**Tissues of expression**	**Receptors**	**Interacting proteins**	**Ref**
S100A1	Zinc- and calcium-binding protein primarily expressed in astrocytes. It binds zinc tightly and weakly binds calcium. Involved in cell proliferation, differentiation, and migration	Brain, heart, muscle, skin, kidney	RAGE, RyR1, RyR2	IFN-a, Annexins	(186)
S100A2	Associated with inflammation and cell migration	Skin, muscle, nervous system, lung, kidney	RAGE, TLR4, AnxA2	FKBP52	(187)
S100A3	Involved in mitochondrial dynamics	Skin, hair cuticle, lung	RAGE, RARα, PML-RARα	PPFIBP1, PGLYRP1, MYH9, AnxA2, TP53, CCR5	(80)
S100A4	Inflammation, cell migration, tumor progression, angiogenesis, apoptosis, and autophagy. It interacts with NMMHC IIA, modulates TP53, and stimulates cytokine production and lymphocyte chemotaxis	Lung, breast, colon, skin, muscle	RAGE	IFN-β, PPFIBP1, PGLYRP1, MYH9, AnxA2, TP53, CCR5	(188)
S100A5	Binds calcium, zinc and copper	Brain	RAGE		(189)
S100A6 (Calcyclin)	Inflammation, cell proliferation, differentiation, reorganization of the actin cytoskeleton and cell motility	Brain, heart, lung, skin, muscle	RAGE	INF-β, CacyBP, Sgt1, AnxA2, TP53	(190)
S100A7 (Psoriasin)	Chemotactic for haematopoietic cells	Fetal ear, skin, tongue	RAGE	RanBP9	(191)
S100A8, S100A9 and S100A8/A9 complex (Calprotectin)	Regulates leukocyte trafficking, neutrophil number and survival, metabolism, pro-inflammatory alarmin, antimicrobial, oxidant scavenger, apoptosis inducer	Myeloid cells, epithelial cells, monocytes, endothelial cells, keratinocytes, macrophages	TLR4, RAGE, CD147, CD69	CEACAM3, tubulin, CD69, CYBA, CYBB	(192, 193)
S100A10	Plasminogen receptor, involved in trafficking membrane protein, act as oncoprotein	lungs, spleen, bone marrow, testis, skeletal muscle etc.		AnxA2	(194, 195)
S100A11 (Calgizzarin)	Cell proliferation, differentiation, and migration	Skin, spleen, lung	RAGE	AnxA1,2,6, HDAC6, TP53, PEX14, RAD51, S100B	(196)
S100A12 (Calgranulin C)	Pro-inflammatory, antimicrobial	Neutrophils, monocytes, epithelial cells	RAGE, TLR4	CacyBP	(197)
S100A13	Involved in non-classical release of IL-1α, FGF-1	Heart, skeletal muscle, lung	RAGE	IL-1α, ProTa, FGF-1, Vimentin	(198)
S100A14	Role in the regulation of cell migration by modulating MMP2	High in colon, low in lung, kidney, liver	RAGE	P53/TP53	(199)
S100A7A (koebnerisin or S100A15)	Antimicrobial in skin and digestive organ	Skin	RAGE		(200)
S100A16	Single Ca^2+^ binding site, inflammation and cell migration	High in esophagus, adipose tissues and colon, low in lung, brain	RAGE	S100A14	(201)
S100B	More affinity to Zn^2+^ than Ca^2+^, neuroinflammation and neuroprotection	Brain, nervous system	RAGE	ATAD3A, S100A6, PPP5C, TPPP	(19)
S100P	Microvilli formation in epithelial cells	Brain, heart, lung, skin, muscle	RAGE	S100A1, S100Z, CacyBP, Ezrin, PPP5C	(202)

The information on salient features, tissues of expression, receptors, and interacting proteins were sourced from protein database of NLM, String, Uniprot, and Protein atlas. AnxA2, ANNEXXINA2; ATAD3A, ATPase family AAA domain containing 3A; CacyBP, calcyclin-binding protein; CCR5, C-C chemokine receptor 5; CEACAM3, carcinoembryonic antigen related cell adhesion molecule 3; CYBA, cytochrome B(558) alpha; FGF-1, Fibroblast growth factor-1; FKBP52, FK506-binding protein 52; HDAC6, histone Deacetylase 6; IFN-β, interferon-beta; IL-1α, interleukin-1 alpha; MYH9, Myosin heavy polypeptide 9; PEX14, peroxisomal biogenesis factor 14; PGLYRP1, peptidoglycan recognition protein 1; PML, promyelocytic leukemia; PPP5C, protein phosphatase 5 catalytic subunit; PPFIBP1, PPFIA binding protein 1; PPP5C, protein phosphatase 5 catalytic subunit; RAD51, RAD51 recombinase; RAGE, receptor for advanced glycation end products; RanBP9, Ran-binding protein 9; RARα, retinoic acid receptor-alpha; RyR1, ryanodine receptor 1; TLR4, toll-like receptor; TP53, tumor protein p53; Sgt1, suppressor of G2 allele of Skp1; TPPP, tubulin polymerization promoting protein.

**Figure 1:**
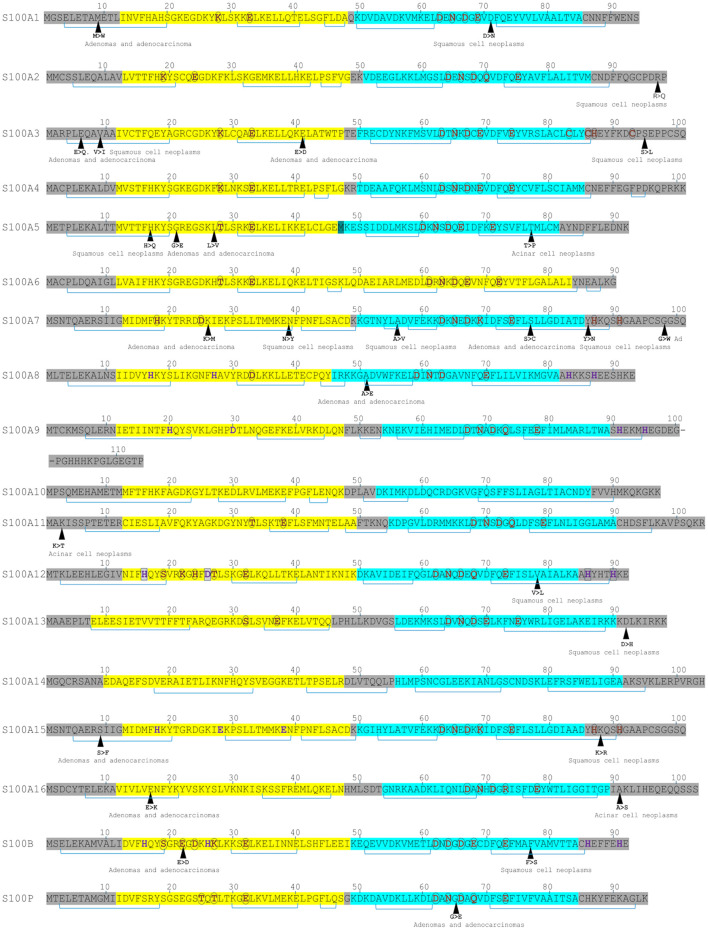
Sequence features of S100 proteins highlighting metal-binding sites and lung cancer-associated mutations. Yellow highlights indicate EF-hand motifs; turquoise highlights, the canonical EF-hand. Brown residues denote Ca^2+^-binding sites; purple residues, Zn^2+^-binding sites; and boxed purple residues, Cu^2+^-binding sites. Brackets represent α-helices. Single-nucleotide mutations associated with lung cancers are indicated.

S100 proteins bind calcium (via EF-hand motifs), as well as zinc and copper ions (6, 16, 17) (Figure 1). Structural analyses show that S100 proteins have at least three active sites on two surfaces, enabling diverse protein interactions for their biological effects, which are often modulated by calcium-induced conformational changes (18). S100 proteins can be categorized into three groups based on their functions: (a) intracellular regulators, (b) dual-function proteins acting intracellularly and extracellularly (19) and (c) primarily extracellular entities (5). Intracellular S100 proteins regulate cell functions like growth, movement, cell cycle, transcription, and differentiation. Extracellularly, they influence inflammation, migration, tissue development, and repair and enhance leukocyte and tumor cell invasiveness (5).

**Figure 2:**
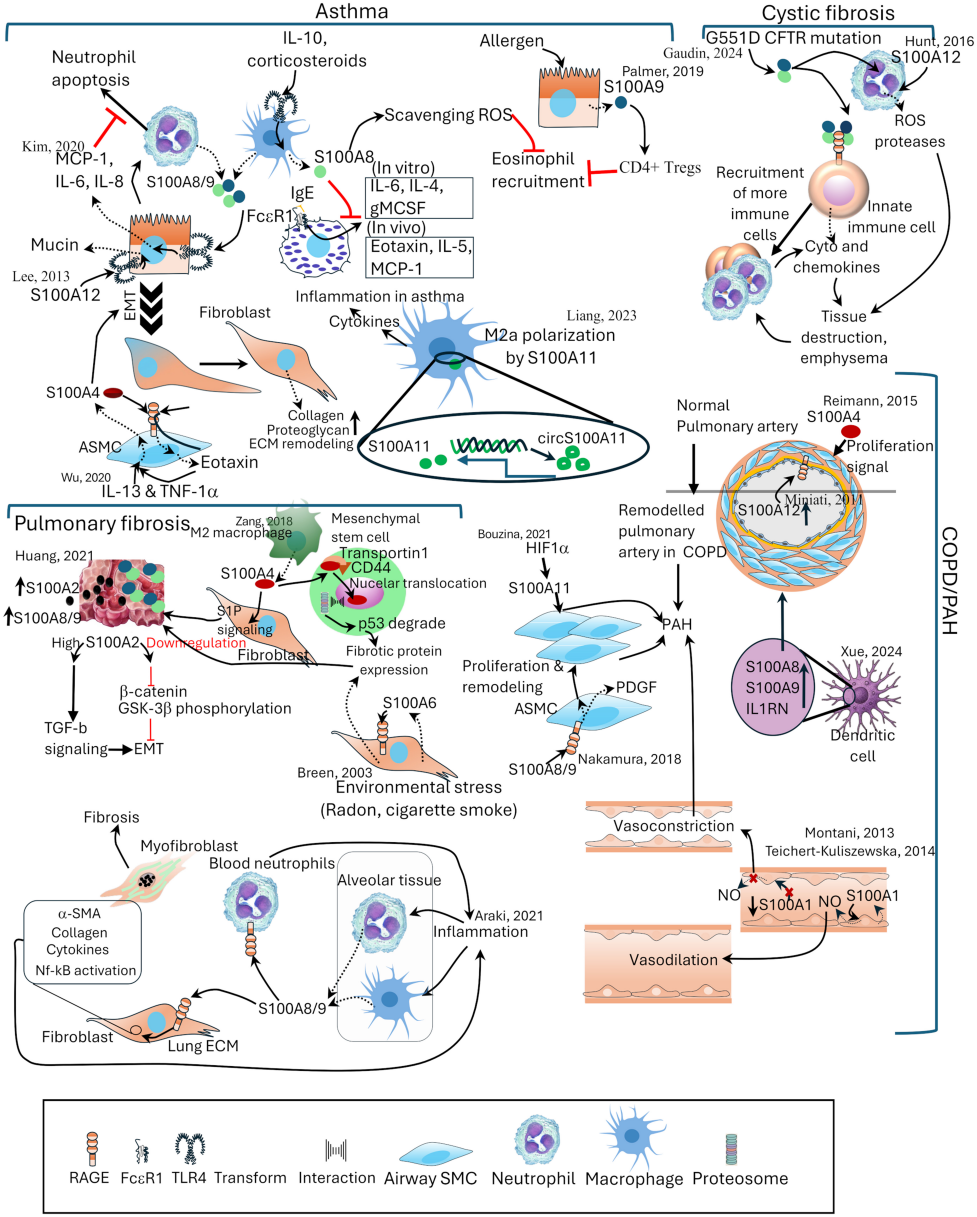
The distinct and shared roles of S100 proteins in lung pathologies. Schematic representation of S100 protein involvement in major lung diseases, including asthma, cystic fibrosis, pulmonary fibrosis, and COPD/pulmonary arterial hypertension (PAH). In asthma, S100A8/A9, S100A4, and S100A11 regulate cytokine production, neutrophil apoptosis, and eosinophil recruitment. In cystic fibrosis, S100A9 and S100A12 contribute to immune cell recruitment, protease release, and emphysematous tissue destruction. In pulmonary fibrosis, S100A2, S100A4, S100A6, S100A8/9, and S100A11 promote fibroblast activation, epithelial–mesenchymal transition, and extracellular matrix remodeling. In COPD/PAH, S100A1, S100A4, S100A8/9, and S100A12 are involved in airway smooth muscle proliferation, vascular remodeling, and vasoregulatory imbalance. Reported interactions with signaling pathways, transcriptional regulators, and environmental stressors are indicated. ECM, extracellular matrix; EMT, epithelial mesenchymal transition; GM-CSF, granulocyte-macrophage colony-stimulating factor; NO, nitric oxide; S1P, sphingosine 1 phosphate; SMC, smooth muscle cell; TGF-β, transforming growth factor- β.

Clinically, dysregulated S100 proteins are valuable diagnostic and prognostic markers in various diseases, including neurodegenerative disorders (20), cardiomyopathy (21), and lung diseases (10) (Figure 2). S100 proteins help in distinguishing between conditions like idiopathic pulmonary fibrosis (IPF) and rheumatoid arthritis-associated interstitial pneumonia (IP) where S100 protein-positive dendritic cells are present only in the latter (22). CD8^+ve^ lymphocytes are more prominent in fibrosing regions surrounding S100-positive dendritic cells than CD4^+ve^ lymphocytes (23). S100A4 and S100B overexpression is associated with poor prognosis and tumor metastasis in lung cancer (10, 20–25) *(see* Table 2 *for roles in lung diseases)*.

**Table 2:** S100 proteins in different lung diseases.

**S100 protein**	**Intra/extracellular (secreted) or both**	**Lung disease/condition**	**Potential diagnostic location of S100 protein**	**Clinical implications**
S100A1	Intracellular	COPD, Pulmonary hypertension	Tissue	Primarily intracellular in cardiomyocytes and smooth muscle cells, linked to pulmonary hypertension (55, 203).
S100A2	Both	Pulmonary fibrosis	Tissue	Elevated in fibrotic lung tissue (78).
		Lung cancer (SCC)	Serum	Increased in tissue, (98) serum (97).
S100A3	Intracellular	Pulmonary fibrosis		Elevated in fibrotic lung tissue (78).
S100A4	Both	Asthma	Sputum	Increased in sputum (204), contributes to airway remodeling and inflammation.
		COPD	Serum	Increased in lung and serum (57)
		Pulmonary fibrosis	BALF	Increased in tissue and BALF, activates fibroblast to myofibroblasts (87).
		Lung cancer	Tissue	Increased in tissue, promotes metastasis in lung cancer by inducing EMT (105).
S100A5	Intracellular	NSCLC	Tissue	Increased mRNA. Correlate with worst prognosis in non-smoking NSCLC (109).
S100A6	Both	Pulmonary Fibrosis	Tissue	Increased in BALF and biomarker for lung fibrosis and vascular damage (85).
		Lung Cancer	Serum	May help to predict lymph node metastasis in ADC (112). Diagnostic marker for early NSCLC (113).
		Acute lung injury	Serum	S100A6 plays a role in airway repair and lung injury after EGFR-TK inhibitor treatment (160).
S100A7	Extracellular	Lung Cancer	Lung tissue	Elevated in cancer tissue, transdifferentiation process from ADC to SCC, poor prognosis (121, 123, 124, 205–207).
S100A8/9	Both	Asthma	BALF, sputum	Elevated levels linked to inflammation during exercise-induced bronchoconstriction (47).
		COPD	Lung tissue, BALF, sputum	Elevated, chronic inflammation, biomarker identification, and disease progression in COPD (60).
		Cystic fibrosis	Sputum, BALF, nasal tissues, mucosa, serum	Elevated levels in children suggest their potential as biomarkers and therapeutic targets (63, 64).
		Pulmonary fibrosis	BALF	Elevated levels are associated with lung fibrosis severity in systemic sclerosis, linked with poor prognosis (91).
		Lung cancer	Sputum, Serum,	May be used as biomarker in smokers with asbestos exposure for early detection of lung cancer (130, 131). Elevated in advanced stages, play role in metastasis, poor prognosis (208).
		COVID-19	Serum	Elevated in lung tissue, mucus hypersecretion, regulates mast cells (172, 209, 210).
		Post lung transplant injury	Plasma	Elevated levels associated with prolonged ischemic times, poorer outcomes, and may be potential therapeutic targets (211, 212).
		Acute lung injury	Lung tissue	Neutrophil recruitment (161).
S100A10		Asthma	PBMC	circS100A11 M2a macrophage activation (53).
		COVID-19	Peripheral blood cells	Associated with inflammation, disease severity, and reduced lymphocyte counts in COVID-19 patients (177).
S100A11	Intracellular	COPD	BALF, sputum and serum	Increased activity promotes inflammation (164).
		PAH	Plasma	S100A11 promotes vascular remodeling (76).
		Lung cancer	Tissue	Increased in ADC and SCC tissues, reduced in SCLC (142). Plays role in chemoresistance, metastasis, poor prognosis (144).
S100A12	Both	Asthma	BALF, sputum	Associated with increase in IgE (213).
		Cystic fibrosis	Airway fluids	Increased expression contributes to inflammation (68).
		ILD	Blood and BALF	Elevated and associated with disease severity (94).
		ARDS		Increased along with sRAGE and HMBG1 (164).
S100A13	Both	Pulmonary fibrosis	Low levels in lungs	Truncated form associated with familial pulmonary fibrosis (79).
		Lung cancer (ADC)	Serum	Associated with poor survival rate, angiogenesis (146). Strong association with metastasis (148), poor survival rate, and angiogenesis (146).
S100A14	Both	Lung cancer	Tissues, serum	Linked with distant metastasis, prognostic marker (152).
S100A15	Both	Lung cancer (ADC)	Tissues, serum	Poor prognosis marker in ADC (214).
S100A16	Both	COPD	BALF, sputum, serum	Elevated; induces cognitive impairment (215).
S100B	Extracellular	Lung cancer (ADC)	Tissue, serum	Early tumor initiation and reduced at late stages (155), promotes brain metastasis (153).
		Covid-19	Serum	Elevated (175).

ADC, adenocarcinoma; ARDS, acute respiratory distress syndrome; BALF, bronchoalveolar lavage fluid; COPD, chronic obstructive pulmonary disease; EMT, epithelial-mesenchymal transition; HMBG1, high mobility group box 1; ILD, Interstitial lung disease; NSCLC, non-small cell lung cancer; PAH, pulmonary arterial hypertension; PBMC, peripheral blood mononuclear cells; SCC, squamous cell carcinoma; sRAGE, soluble RAGE.

Despite these findings, the collective literature on S100 proteins in lung diseases remains limited, including their roles in COVID-19 and lung transplantation. This review aims to provide a comprehensive exploration of the diagnostic, prognostic, and therapeutic potential of S100 proteins in these contexts, offering a detailed analysis to bridge existing knowledge gaps.

## Metal ion binding and conformational changes of S100 proteins for intra- and extracellular functions

The EF-hand motif of many S100 proteins have Zn^2+^/Cu^2+^ binding sites in addition to Ca^2+^ metal ions(26–30). This unique feature provides them the versatility of performing both intracellular and extracellular functions (26, 31, 32).

## The Ca^+^ switch for intracellular functions

The EF-hand motif of S100 proteins binds Ca^+^ ions, triggering a conformational change often described as the “S100 Ca^+^-switch.” This structural rearrangement exposes previously buried hydrophobic surfaces, creating docking sites for a wide array of intracellular targets such as enzymes, cytoskeletal proteins, and transcriptional regulators (33, 34). Through these interactions, S100 proteins regulate fundamental cellular processes, including proliferation, differentiation, apoptosis, and motility. Thus, Ca^+^-dependent conformational dynamics are central to the intracellular signaling roles of S100 proteins (8, 19).

## Transition metal-dependent structural rearrangements for extracellular functions

In addition to Ca^+^ binding, S100 proteins possess unique transition metal-binding sites at their dimer interface, particularly for Zn^+^ and Cu^+^ (27–29, 33). Binding of these metals induces structural changes distinct from those caused by Ca^+^ (35, 36). These rearrangements enable S100 proteins to interact with cell surface receptors, most notably the receptor for advanced glycation end products (RAGE) and toll-like receptor 4 (TLR4). These interactions mediate extracellular signaling through both autocrine and paracrine pathways, which connects them to regulation of the immune system, inflammation, and many diseases.

## Distinction of S100 proteins from other EF-hand proteins

While classical EF-hand proteins like calmodulin also undergo Ca^+^-induced conformational changes, S100 proteins stand out due to their dual/triple metal-binding capability and the resulting distinct conformational responses (37, 38). Ca^+^ binding exposes hydrophobic pockets for intracellular interactions, whereas Zn^+^/Cu^+^ binding at the dimer interface enables extracellular receptor engagement (5, 26, 39). This adaptability allows S100 proteins to serve as both intracellular regulators and extracellular signaling molecules—an evolutionary specialization not shared by simpler Ca^+^ sensors (26, 39).

The structural plasticity of S100 proteins, governed by their ability to bind multiple metal ions, underpins their dual roles. By coupling Ca^+^-induced conformational changes to intracellular signaling and Zn^+^/Cu^+^-induced rearrangements to extracellular receptor interactions, S100 proteins uniquely bridge intracellular regulation with extracellular communication (39, 40). This property sets them apart from other EF-hand proteins and explains their prominent involvement in processes ranging from cytoskeletal dynamics to cancer metastasis and inflammation (39).

## S100 proteins induce inflammation and airway remodeling in asthma

Asthma is a chronic inflammatory disease of the airways characterized by bronchoconstriction, elevated levels of allergen-specific IgE, airway hyperresponsiveness and remodeling (41). Until now, S100A4, S100A8/S100A9 (calprotectin), S100A11, and S100A12 have been implicated in the pathophysiology of asthma, exhibiting both similarities and differences in their mechanisms of action.

S100A4, also known as fibroblast-specific protein 1 (FSP1), contributes to asthma by promoting inflammation and epithelial-mesenchymal transition (EMT) in the airway (42). Similarly, in pleural fibrosis, S100A4 has been demonstrated to stimulate the production of transforming growth factor-β (TGF-β) and facilitate epithelial-mesenchymal transition (EMT) in pleural mesothelial cells (43). While this specific mechanism has not been investigated in asthma, it is plausible that S100A4 plays a similar role in the airway remodeling observed in asthmatic patients. Notably, during episodes of exacerbated inflammation, cytokines such as IL-13 and TNF-α trigger the release of S100A4 from airway smooth muscle cells. The secreted S100A4 subsequently engages the RAGE, thereby activating the Akt/NF-κB signaling pathway (44). This activation results in the synthesis of eotaxin and further production of S100A4, consequently establishing a positive feedback loop that could perpetuate inflammation in individuals with asthma. Diagnostically, elevated levels of S100A4, like calprotectin, in the sputum of asthmatic patients correlate with airway hyperresponsiveness, providing evidence of its role in disease exacerbation. Thus, S100A4 neutralizing antibodies have shown promising results of reducing airway hyperresponsiveness and inflammation and preventing fibrosis in animal models (42).

The S100A8/A9 heterodimer plays a dual role in asthma pathogenesis, depending on the inflammatory milieu and asthma subtype. During infection and inflammation, extracellular S100A8/9 levels rise and engage TLR4 on bronchial epithelial cells, activating MAPK and NF-κB pathways to induce neutrophil survival cytokines such as MCP-1, IL-6, and IL-8 (45, 46), thereby intensifying airway inflammation. Aligned to that, elevated S100A8/A9 levels are observed in the serum and sputum of asthmatic patients, particularly during episodes of exercise-induced bronchoconstriction (47), without any difference between the subgroups of asthma or compared to COPD (48, 49). In addition, elevated expression of S100A8/9 was observed in lungs of mouse model of asthma, a finding that aligns with observations in human asthma patients. In these patients, calprotectin levels were associated with several clinical parameters, including the ratio of forced expiratory volume in one second to forced vital capacity, smoking history, body mass index, and the percentage of neutrophils in the blood (49). In contrast, in allergic, Th2-driven asthma, S100A8/A9 exerts a regulatory function. In wild-type mice, *Alternaria alternata* challenge augmented S100A8/A9 release into the alveolar space and elevated its expression in the epithelium. Compared to wild-type, S100A9-deficient mouse model displayed severe airway inflammation, marked by elevated IL-13, CCL11, CCL24, serum IgE, eosinophil recruitment, and increased airway resistance and elastance. The study suggests S100A9-mediated protection occurs via regulation of CD4+ T CD25^low^ regulatory T (Treg) cells (50). However, S100A9 levels in sputum are seen higher in neutrophilic uncontrolled asthma patients compared to controlled asthma cases (51). A therapeutic potential for S100A9 was demonstrated in rats by significantly reducing isometric tension of isolated tracheal spirals (52). This dual functionality underscores its context-specific nature, acting as an inflammatory amplifier in innate immune settings and a modulator in adaptive, allergic responses, with its net impact depending on the prevailing immunological profile of the disease.

S100A11 has an immunomodulatory effect in asthma. S100A11-gene derived circular RNA (circS100A11) is significantly higher in monocytes of pediatric asthma patients. circS100A11 enhances S100A11 expression that promotes STAT6-mediated M2a macrophage activation and exacerbates lung inflammation in mouse model (53). However, an airway smooth muscle cell (ASMC) relaxing effect by S100A11 is also reported in an allergen-induced asthma model (54). Recombinant S100A11 treatment in OVA-challenged rat results in a reduced airway hyperresponsiveness (AHR), and it reduces acetylcholine-induced myosin light chain phosphorylation in ASMC, in a calcium-independent manner. It denotes there may be cell-type specificity existing in response to S100A11 (54). Whether S100A11 has any impact on mast cells, histamine release or any other broncho-constrictive pathways still need to be addressed. The role of S100A11 in promoting inflammation to ward off infections/allergens while also providing a compensatory relaxation effect in ASM cells underscores the complexity of S100 proteins in asthma and their potential as targets for nuanced therapeutic strategies (53, 54).

S100A12, as well as S100A8 and S100A9, was shown to activate TLR4 and RAGE in normal bronchial epithelial cells and lung carcinoma cells *in vitro* to produce MUC5AC, a predominant protein in mucin (51). Since mucin production is a common feature in severe asthma, this observation underscores the importance of these S100 proteins in airway congestion, and their regulation could be of therapeutic value.

It is evident that S100 proteins contribute to inflammation and remodeling in asthma, often via RAGE and TLR4, yet vary in cellular targets and mechanisms. Diagnostically, they may serve as markers of severity and phenotype; prognostically, they could predict progression in severe asthma.

## S100 proteins increase chronic inflammation in COPD

COPD is a progressive disease marked by persistent airflow limitation due to neutrophilic airway inflammation, emphysema, and vascular remodeling. S100 proteins play critical roles in both the inflammatory and structural components of COPD. Serum levels of S100A1 distinguish cachectic COPD patients from non-cachectic ones, establishing it as a biomarker for COPD progression, particularly in the context of cachexia (55).

Increased S100A4 levels in the remodeled intrapulmonary arteries may be an indication of this protein's involvement in vascular remodeling of COPD patients (56). Likewise, elevated S100A4 levels in the serum in conjunction with sphingosine 1 phosphate (S1P) correlate with reduction in lung function (57).

The predominant role of S100A8/9-mediated RAGE activation in COPD is evident from the observation that lower levels of S100A8/9 in RAGE-deficient mice result in decreased cigarette smoke-induced inflammation (58). Chronic inflammation, reduced lung function (59), and IL-17-related signaling in COPD are linked to upregulated S100A8 and S100A9 or their heterodimer in dendritic cells (60). Additionally, increased S100A8/A9 levels in smokers with COPD indicate their potential as biomarkers for diagnosis and tracking disease progression (61).

Elevated S100A12 levels in the airways and blood are associated with poor prognosis in COPD, making it a potential biomarker for disease progression (62). S100A12 effect is mediated through RAGE, while its soluble form, sRAGE, functions as a decoy receptor that limits the inflammation. Low sRAGE levels are linked to severe emphysema and chronic cor pulmonale, promoting the activation of neutrophils and macrophages and contributing to tissue damage.

## S100 proteins regulate neutrophil-mediated inflammation in cystic fibrosis

Cystic fibrosis (CF) is characterized by chronic neutrophilic inflammation and progressive lung damage due to mutations in the CFTR gene. S100 proteins, particularly calprotectin and S100A12, play critical roles in sustaining this inflammation. A marked increase in exocytosis of S100A8/A9 in the airways of CF patients contributes to the perpetuation of neutrophilic inflammation (63, 64). The G551D CFTR mutation leads to dysregulated calcium signaling, which in turn activates S100A8/A9 and promotes the release of pro-inflammatory cytokines. These proteins drive neutrophil degranulation, resulting in the release of proteases and reactive oxygen species (ROS), which cause damage to the airway epithelium and exacerbate lung injury. Elevated levels of S100A8 associated with hyperactive immune response have been observed in experimental models of CF (65, 66).

Coupled with a deficiency of sRAGE (67), increased levels of S100A12 in the airways interact with RAGE, followed by activation of the p38 MAPK pathway in neutrophils leading to the continuous release of pro-inflammatory mediators, contributing to chronic inflammation, worsening CF progression, and impaired lung function (11, 67, 68).

## S100 proteins regulate vascular remodeling in pulmonary arterial hypertension

Pulmonary arterial hypertension (PAH) is characterized by increased pulmonary artery pressure due to vascular remodeling, which results in right heart failure (69). S100 proteins have been implicated in the regulation of vascular homeostasis and remodeling in PAH.

Vascular endothelium-derived S100A1 regulates vascular effects by influencing nitric oxide (NO) production (70, 71). Reduced lung endothelial S100A1 levels may diminish NO expression, which leads to pulmonary vasoconstriction and potentially to PAH (72). The therapeutic potential of S100A1 in PAH was demonstrated by the administration of exogenous S100A1 to S100A1 knockout (KO) mice, leading to improvements in pulmonary artery pressure, vascular resistance, and endothelial cell survival (73).

S100A8/A9 also contributes to vascular remodeling in PAH by promoting smooth muscle cell proliferation and migration. Through RAGE signaling, S100A8/A9 enhances the expression of pro-inflammatory cytokines and growth factors, including PDGF, which accelerates the pathogenesis of pulmonary vascular remodeling (74).

Elevated levels of S100A11 are observed in the plasma of PAH patients (75). Under hypoxic conditions, hypoxia-inducible factor 1-α (HIF-1-α) induces upregulation of S100A11 mRNA in rat lungs, along with increased taurine levels. Administration of taurine attenuates HIF-1-α-induced transcriptional activation of S100A11, suppressing vascular remodeling. This suggests that S100A11 is a potential therapeutic target for vascular remodeling in pulmonary diseases and that taurine could be a treatment to inhibit hypoxia-induced vascular remodeling (76).

## S100 proteins mediate EMT in pulmonary fibrosis

Pulmonary fibrosis (PF) is characterized by the excessive deposition of extracellular matrix (ECM) components and progressive scarring of lung tissue (77). Several S100 proteins, notably S100A2, S100A3, S100A4, S100A6, S100A8/A9 and S100A13 are deeply implicated in the mechanisms underlying fibrotic progression.

Elevated levels of S100A2 are found in lung tissues of PF patients. Its downregulation inhibits TGF-β1-induced EMT by blocking β-catenin expression and GSK-3β phosphorylation in A549 cells. Lithium chloride, a Wnt/β-catenin pathway activator, reverses EMT inhibition caused by S100A2 silencing, suggesting a potential treatment for PF by the inhibition of S100A2 (78).

**Figure 3:**
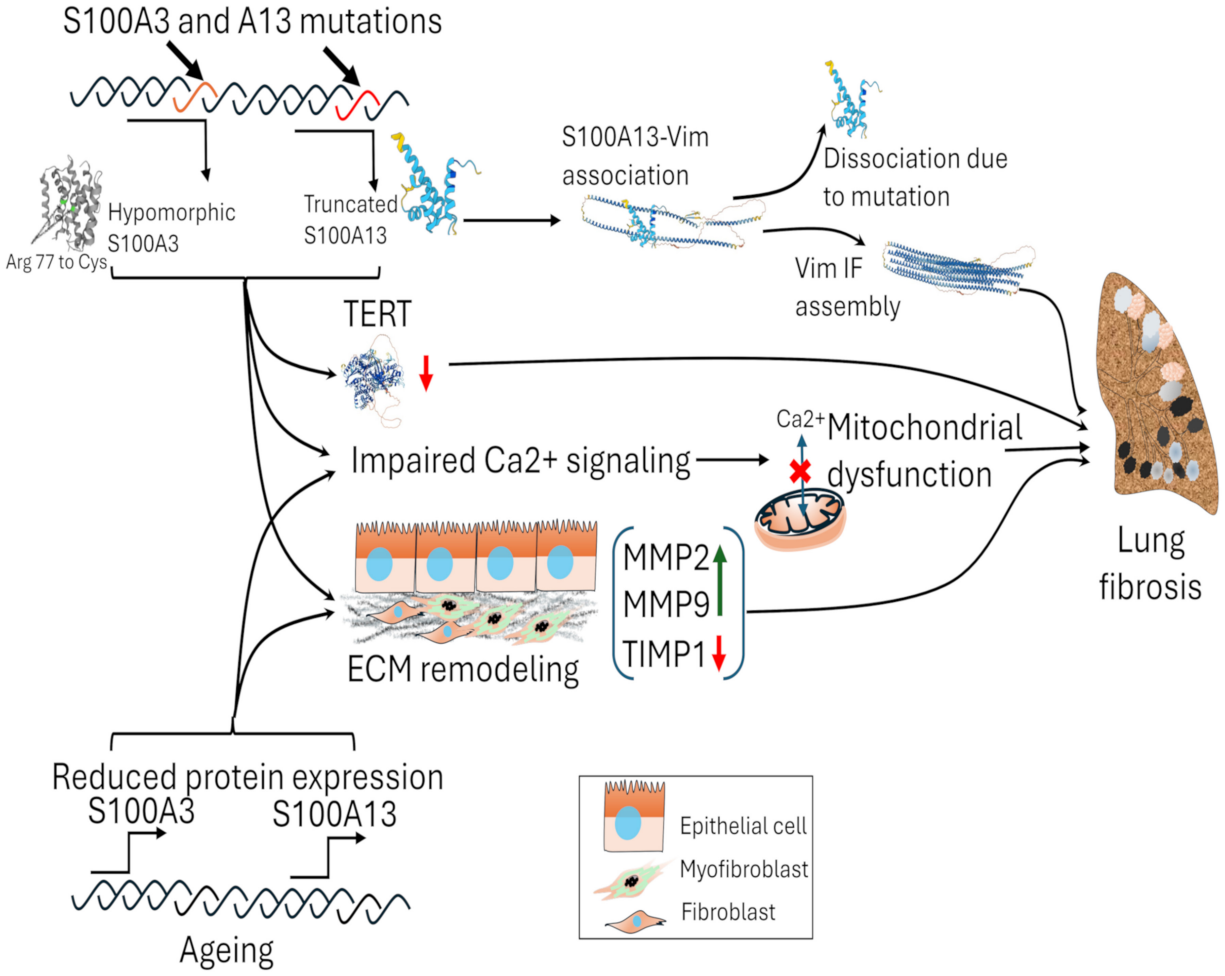
The impact of S100A3 and S100A13 mutations and the reduction of wild-type S100A3 and S100A13 while aging on lung fibrosis. Mutations in S100A3 (c.229C > *T*; Arg 77 to Cys) and S100A13 (c.238–241delATTG) proteins lead to functional alterations, affecting calcium signaling and telomerase reverse transcriptase (TERT) expression. The mutant variants of S100A3 and S100A13 affect Ca^2+^ signaling, mitochondrial dysfunction, and ECM remodeling by the increased expression of MMPs and decrease in TIMP1. S100A13 interacts with vimentin intermediate filaments (IF), but mutations cause dissociation, leading to defects in vimentin IF assembly. These genetic changes contribute to mitochondrial dysfunction and tissue damage. In sporadic cases of pulmonary fibrosis, age-related declines in S100A3 and S100A13 protein expression can contribute to susceptibility to developing pulmonary fibrosis. TERT, telomerase reverse transcriptase; Vim IF, vimentin intermediate filament.

S100A3 and S100A13 mutations are particularly relevant in the context of familial early-onset pulmonary fibrosis (PF), with our research showing that these mutations disrupt key cellular processes that contribute to fibrosis (Figure 3). S100A3 mutations impair calcium signaling, disrupting cellular homeostasis, while S100A13 mutations affect mitochondrial function and cytoskeletal dynamics via vimentin, driving early fibrotic changes. These dual disruptions in S100A3 and S100A13 affect both intracellular and extracellular processes essential for fibrosis. Our findings suggest that targeting these proteins, or their downstream effects, could help reverse the defective signaling pathways and provide therapeutic benefit in familial PF cases, potentially preventing excessive fibrotic remodeling (79–82).

M2 macrophage-released S100A4 activates lung fibroblasts through sphingosine 1 phosphate (S1P) signaling pathway to drive fibrosis (83–85). Nuclear translocation of S100A4 by making a complex with CD44 and transportin1 enhances the fibrogenic potential of mesenchymal progenitor cells. The nuclear S100A4 interacts with the proteasome to degrade p53 is crucial in fibrogenesis (86). *In vivo* studies have demonstrated that S100A4 deficiency protects against pulmonary fibrosis, consistent with its abnormal increase in human IPF (87).

S100A6 plays a major role in maintaining lung integrity by involving itself in tissue repair and fibroblast proliferation in response to mechanical stress (88, 89). S100A6 is elevated in BALF samples from PF-systemic sclerosis patients compared to smoker and non-smoker controls (85). The interaction between S100A6 and RAGE plays a vital role in mediating inflammatory and oxidative damage from prolonged cigarette smoke or radon exposure. This underscores S100A6 as a potential biomarker and therapeutic target against environmental-induced lung damage.

Elevated S100A8/A9 expression in lung, BALF and blood is correlated with the severity of PF-systemic sclerosis patients as well as sarcoidosis (90, 91). The main sources of S100A8/9 in the lung are macrophages and neutrophils. Upon an inflammatory signal, they release S100A8/9, which is released into the lung ECM and blood. The fibroblasts in the ECM get activated via RAGE and transdifferentiate into myofibroblasts. The expression of pro-inflammatory cytokines, collagen, and α-SMA are all found elevated and associated with myofibroblast formation (92). Moreover, during acute exacerbations of IPF, increased serum S100A8/A9 concentrations are linked to poor prognostic outcomes and reduced survival, proposing their use as prognostic markers. Exposure to zinc oxide nanoparticles can elevate respiratory S100A8 and S100A9 levels, potentially increasing lung inflammation and exacerbating fibrotic and cancerous conditions (93).

Elevated S100A12 levels in blood and BALF of patients with idiopathic interstitial pneumonias (IIP) and IPF are associated with disease severity and can be used as prognostic markers, particularly in IPF, where higher levels indicate a poorer prognosis (94). S100A12 inhibits physiological fibroblast migration for tissue repair through RAGE-p38 MAPK signaling. Targeting the S100A12-RAGE-p38 MAPK pathway could be beneficial for pulmonary disorders with abnormal tissue remodeling (95).

In pulmonary fibrosis, S100 proteins collectively drive inflammation, fibroblast activation, and ECM deposition, often via RAGE-mediated pathways. However, there are notably divergent roles among them; for example, S100A4 and S100A6 directly promote fibroblast activity and remodeling, while S100A8/9 and S100A12 amplify inflammation and serve as prognostic markers. S100A2 uniquely regulates EMT. However, normal function of S100A3 and S10013 appears to be important for normal physiology of lungs, and certain mutations in S100A3 and S100A13 contribute to familial PF. On the other hand, S100A6 responds to environmental triggers and leads to its abnormal expression leads to fibrogenesis. These contrasting functions underscore the complexity of S100 proteins in PF and their promise as tailored diagnostic and treatment targets.

## S100 proteins in lung cancer

**Figure 4:**
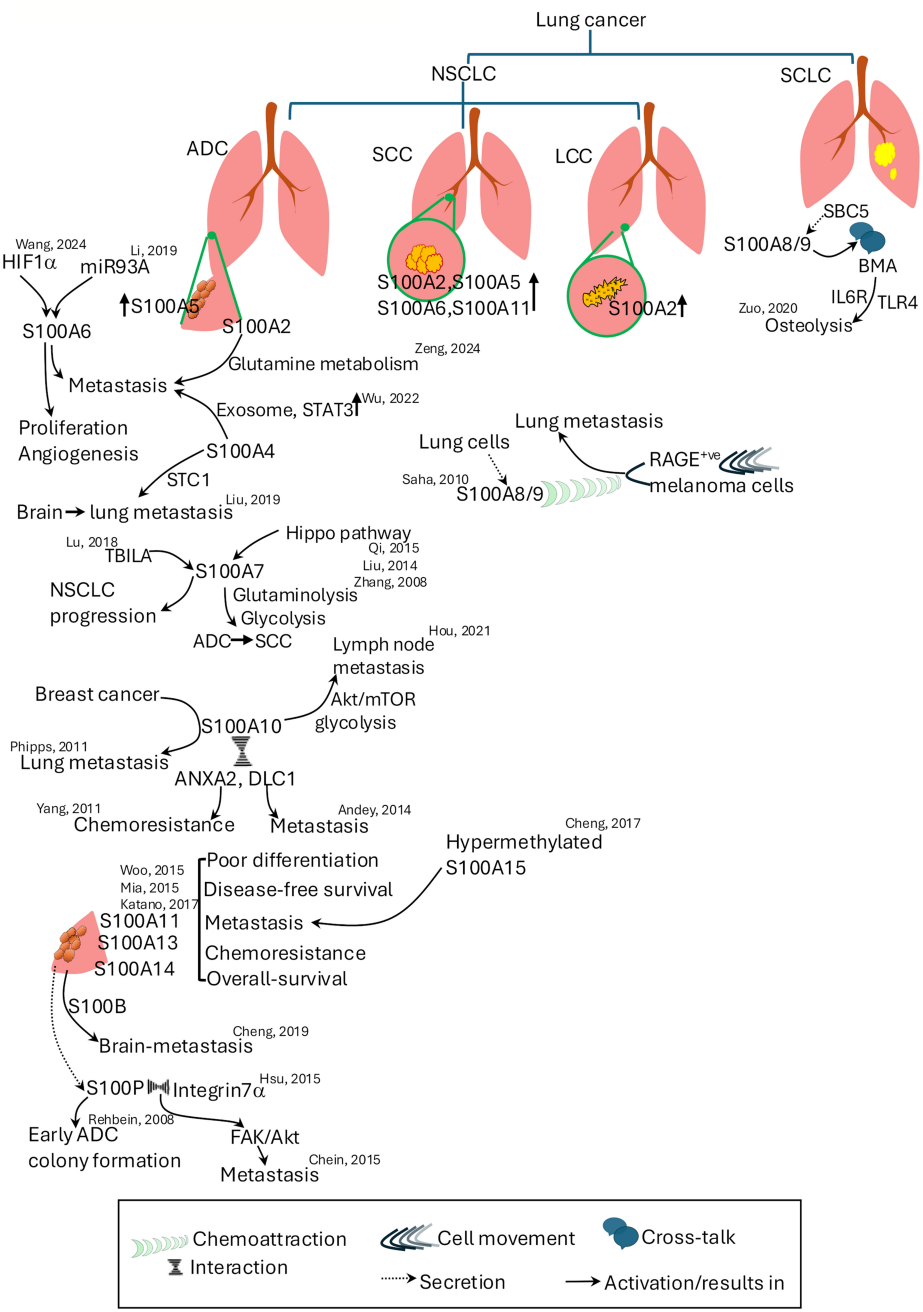
Roles of S100 proteins in lung cancer subtypes and metastasis. S100 proteins contribute to various aspects of lung cancer progression, metastasis, and chemoresistance. The figure illustrates the subtype-specific expression and functions of S100 proteins in non-small cell lung cancer Key roles include regulation of proliferation, angiogenesis, metabolic reprogramming, and metastatic dissemination. Cross-talk between S100 proteins and other signaling pathways, including Hippo, Akt/mTOR, STAT3, and RAGE-mediated mechanisms, is shown. Specific S100 proteins associated with metastasis to brain, lymph nodes, and lungs are highlighted. References denote supporting studies. ADC, adenocarcinoma; ANXA2, annexin A2; Akt, Ak strain transforming; BMA, bone marrow adipocytes; DLC1, deleted in liver cancer 1; FAK, focal adhesion kinase; HIF1-α, hypoxia inducing factor 1–α; LCC, large cell carcinoma; mTOR, mammalian target of rapamycin; NSCLC, non-small cell lung carcinoma; SBC5, small cell carcinoma-5; SCC, squamous cell carcinoma; SCLC, small cell lung cancer; STC1, stanniocalcin 1; TBILA, TGFβ-induced lncRNA; TLR4, toll-like receptor 4.

Lung cancer, particularly non-small cell lung carcinoma (NSCLC), is a heterogeneous disease encompassing various subtypes, each characterized by distinct molecular and clinical features (96). The S100 proteins, present primarily in NSCLC and its early-stage expression significantly influence tumor progression and therapy resistance, emerging as potential biomarkers and therapeutic targets in disease management. Their specific role in small cell lung carcinoma (SCLC) is limited and, in some cases, yields negative results. A comprehensive figure capturing the roles of S100 proteins in lung cancer is provided in Figure 4.

Elevated levels of S100A2 in the serum of NSCLC patients serve as a potential diagnostic and prognostic biomarker, especially in early-stage disease and development of metastasis (97–99). Lung adenocarcinoma (ADC), squamous cell carcinoma (SCC), large cell carcinoma, and atypical carcinoids show high S100A2 expression, while small cell lung carcinoma (SCLC) lacks S100A2 expression (100). Studies reveal that TFAP2A, a transcriptional regulator, increases S100A2 expression, a distinct molecular marker for pre-invasive stages of ADC (101) and this elevation contributes to ADC metastasis by regulating glutamine metabolism (102). Although S100A2 mutations that can be attributed to NSCLC are rare, alterations in the gene have been identified in lung SCC samples (103). Even though S100A3 does not have a direct effect on pathogenesis of lung cancer, it alters the response of lung cancer cells to all-trans retinoic acid (ATRA) treatment by interacting with retinoic acid receptor-alpha (RARα) transcription factor, which results in the degradation of RARα and promyelocytic leukemia (PML)-RARα receptor (104).

High expression of S100A4 facilitates NSCLC metastasis and immunosuppression via exosomes and the STAT3 pathway, which results in poor tumor differentiation, inhibition of autophagy, and worse prognosis (105, 106). S100A4 enhances breast-to-lung metastasis through stanniocalcin 1 (STC1). Inhibiting S100A4 reduces STC1-induced metastatic colonization, indicating its promise as a therapeutic target (107). S100A4 influences lung cancer cell metabolism by regulating mitochondrial function and oxygen consumption, with reduced levels promoting a shift to glycolysis and less aggressive behavior (108).

Increased expression of S100A5 mRNA has been noted in NSCLC, and it is correlated with worse prognosis in non-smoking NSCLC patients (109). Bioinformatic analysis of TCGA-derived lung SCC data identified S100A5 as a key immune-related differentially expressed gene (DEG) for constructing a prognostic model. Integration of S100A5 with ten other genes enables effective prognosis assessment, and this model offers insights for personalized immunotherapy and improved diagnostic strategies for SCC (110).

S100A6 signaling through RAGE may be involved in lung cancer pathogenesis (111), and it is a promising diganostic marker, like S100A2, for early stage NSCLC detection. Its differential expression distinguishes NSCLC from SCLC, correlating with advanced stages and metastasis in lung ADC (112) and worse outcomes in older SCC patients and poorly differentiated tumors (113–115). Hypermethylation of S100A6 promotor confers radiation resistance in NSCLC cell line H1299 (116). Overexpression of S100A6, driven by miR-193a (117) or by HIF-1-α-induced hypermethylation (118) of the S100A6 promoter region, has been linked to the promotion of lung cancer cell proliferation, invasion, migration, and angiogenesis. However, a study suggests that S100A6 expression and its post-translational modifications correlate with improved outcomes in stage 1 NSCLC patients, especially in tumors without p53 expression, suggesting a pro-apoptotic role and potential interactions with p53 (119).

S100A7 act as metabolic regulator in lung ADC (120), driving glycolytic and glutaminolytic pathways, and Hippo pathway-mediated overexpression of it accelerates trans-differentiation from lung ADC to SCC and is associated with poor prognosis (121–123). Silencing S100A7 reduces proliferation, NF-κB activity, and proliferation in lung cancer cells (122, 124). TGFβ-induced lncRNA (TBILA) activates the S100A7-JAB1 signaling pathway, which plays a critical role in regulating the cell cycle and contributes to the progression of NSCLC (125).

S100A8/A9 plays a role in metastasis, as shown in SBC5 (small cell lung carcinoma cell line) invasion via the S100A8/A9-IL6R-TLR4 pathway, a key mechanism facilitating osteolytic activity in bone metastases (126). RAGE-expressing melanoma cells are chemotactically attracted by S100A8/A9 to lung (127). In NSCLC, S100A8, S100A9, and S100A12 proteins serve as potential biomarkers and assist in monitoring therapeutic responses (128, 129). Elevated S100A8 and/or S100A9 levels in male NSCLC and subtype patients, smokers, and those with advanced disease correlate with survival outcomes, suggesting their potential as prognostic markers (130–132). Increased plasma S100A8 levels in NSCLC patients with venous thromboembolism (VTE) suggest its use as a biomarker for VTE diagnosis (133).

Elevated levels of S100A10 are associated with advanced cancer progression, lymph node metastasis, and poor prognosis in lung cancer types, particularly in ADC and SCC (134, 135) attributed to its role in enhanced cell proliferation, invasion via the Akt-mTOR pathway, and increased glycolysis (136). In breast cancer, elevated S100A10 corresponds to lung metastasis, especially the aggressive triple-negative subtype, as supported by both human data and S100A10-deficient mouse models (137, 138). Mechanistically, the interaction of S100A10 with tumor suppressor DLC1 facilitates metastasis, while its binding with AnxA2 contributes to chemotherapy resistance (139, 140). Additionally, co-elevated levels of S100A10, fibronectin, and tenascin-C in lung tumor ECM highlight their potential as a combined biomarker for predicting patient survival (141).

In ADC and SCC, elevated S100A11 expression in patient lung tissues and serum is associated with poor differentiation, KRAS mutations, shorter disease-free survival (142), advanced tumor stages and metastasis (143), and chemoresistance, as reducing its expression sensitizes cancer cells to chemotherapy like cisplatin (144). In contrast to NSCLC, the expression of S100A11 is low in SCLC (145).

Elevated expression of S100A13 in early-stage NSCLC is associated with poorer overall survival and disease-free survival rates. It contributes to enhanced angiogenesis within tumors, promotes invasive behavior of lung cancer cells, and serves as a potential prognostic marker, with higher levels observed in more aggressive cancers (146, 147).

Analyses of lung ADC cases have shown frequent upregulation of S100A14 in tumor tissues and serum correlating strongly with poor differentiation, metastasis, advanced disease stage, smoking history, EGFR mutations, and unfavorable patient outcomes (148, 149). Murine studies have also confirmed that S100A14 is involved in lung metastasis, and *in vivo* knockdown reaffirms its metastasis-promoting effects (150).

S100A15 has gained attention as an important biomarker in lung cancer progression and prognosis, particularly in lung ADC. Analysis of 178 lung cancer specimens revealed that increased nuclear S100A15 expression is associated with distant metastasis and reduced survival in patients on first-line therapy and predicting three-year mortality (151). Hypomethylation of the S100A15 promoter at sites −423 and −248 correlates with disease progression and decreased one-year survival (151). S100A15 also modulates immune response in NSCLC. Upregulation of S100A15 alongside DOK2 in patients pre- and post-chemotherapy identifies it as a potential biomarker for tumor staging and prognosis (152).

High serum S100B levels are proposed as a sensitive biomarker for early detection of brain metastasis in lung ADC (153, 154), promoting proliferation, migration and invasion inhibiting apoptosis as seen in the PC14/B cell line.

S100P plays a stage-dependent and context-dependent role in lung cancer as observed from two different studies. Rehbein et al. (155) report lung ADC expresses S100P in early/T1 stage, but not in advanced/T2 stage, suggesting early tumor initiation rather than aggressive growth in advanced stages. Overexpression of S100P in H358 cell lines promoted colony formation but paradoxically reduced proliferation and migration. Moreover, S100P expression was found to regulate itself by transcriptional feedback (155). In contrast, Hsu et al. (156) report S100P as a pro-metastatic oncogenic driver in lung cancer. S100P promotes migration, invasion, EMT, and metastasis via integrin α7 and downstream FAK/AKT/Src/ZEB1 signaling. Chein et al. (157) also suggest metastatic potential of S100P as Keap1 mediated reduction in S100P levels and decreases metastasis of NSCLC cells. It was also noted that knocking down S100P expression by shRNA in NSCLC animal models reduced angiogenesis and metastasis (158). S100P along with GATA3 and napsin A expression help to distinguish lung-derived bladder adenocarcinoma from primary bladder adenocarcinoma (159).

## S100 proteins in acute lung injury (ALI) and acute respiratory distress syndrome (ARDS)

ALI and ARDS are conditions characterized by the rapid onset of inflammation and damage to lung tissue, leading to impaired gas exchange and respiratory failure. S100 proteins play critical roles in modulating release of proinflammatory cytokines, inflammatory pathways and neutrophils and macrophages responses during these lung injuries.

In ALI, S100A6 is involved in airway epithelial recovery and may affect inflammation and lung damage following EGFR-tyrosine kinase inhibitor treatment (160). Upregulation of S100A6, S100A8, and StefinA3 during airway epithelial repair with gefitinib treatment can increase neutrophil retention, worsening ALI (160).

ALI highlights the role of S100A8/A9 in neutrophil recruitment via TLR4 pathways in alveolar epithelial cells (161). While both proteins can influence neutrophil influx and inflammation, the heterodimer S100A8/A9 exhibits distinct effects. S100A9 promotes mild inflammation through mast cell degranulation and chemokine upregulation, but unlike S100A8, does not induce proinflammatory mediators. Both S100A8 and S100A9 can reduce neutrophil influx in LPS-induced lung injury, potentially through shared mechanisms like sirtuin-1 activation and STAT3 signaling. These findings highlight the distinct roles of S100A8, S100A9, and their heterodimer in lung homeostasis (162).

Elevated levels of S100A12 in BALF and pulmonary tissue suggest its association with neutrophil activation and inflammation. Proinflammatory effects of S100A12 are likely mediated through its interaction with the RAGE receptor, contributing to endothelial activation and further exacerbating lung injury (163). In ARDS, patients exhibit elevated sRAGE, HMGB1, and S100A12 levels, with decreased esRAGE and AGEs. These changes in RAGE isoforms and ligands, including S100A12, differentiate ARDS patients, suggesting a potential role of the RAGE/S100A12 axis in the disease process (164). S100A12 levels in BALF offer promise in distinguishing ARDS from conditions like CF and COPD (165).

## Role of S100 proteins as biomarkers in COVID-19 and long COVID

Elevated mRNA expression of S100A6, S100A8, S100A9, and S100P, have been identified in the nasal swabs of COVID-19 patients. They also identified thioredoxin significantly upregulated in those patients. Thioredoxin inhibitor Auranofin has been found effective to mitigate SARS-CoV-2 replication in hamster model. However, a relationship between S100 proteins and thioredoxin was not elucidated in this study (166). S100A8/A9 is most predictive of severe disease and long COVID, driving cytokine storms and chronic inflammation via TLR4/RAGE (167). In severe COVID-19, elevated S100A8/9 levels drive emergency myelopoiesis, leading to the generation of immature neutrophil subsets and resulting in dysfunctional innate immune responses (168, 169). S100A8/9 can activate these immature neutrophils, and macrophages via TLR4 to induce the production of IL-6, TNF-1α, and S100A8 itself in a positive feedback loop to sustain this cycle of events (169). It has been shown that S100A8/A9 induces IL-8 release from bronchial cells and triggers pro-inflammatory responses in endothelial cells (170). High serum levels of S100A8/A9 in patients at hospital admission correlate with poor outcomes and predict severe disease (171). Transcriptomic analyses have shown overexpression of S100A8, S100A9, S100P and S100A12 in lung tissue from fatal COVID-19 patients (172, 173). S100B levels are also found significantly higher in 38% of ICU admitted COVID-19 patients without any clinical evidence of brain injury. It was also higher in patients succumbed to death compared to those who survived. S100B levels in those patients were correlated with IL-6 levels, illness severity and lymphocyte count. However, the exact cellular source of S100B in these patients remains elusive (174, 175). Tissue hypoxia, critical illness and systemic inflammation may be activating/injuring glial cells to secrete S100B (176). Additionally, the levels of S100A4, S100A9, and S100A10 have been shown to influence inflammation and disease severity, associating them with ALI and reduced lymphocyte counts in COVID-19 patients (177).

**Figure 5:**
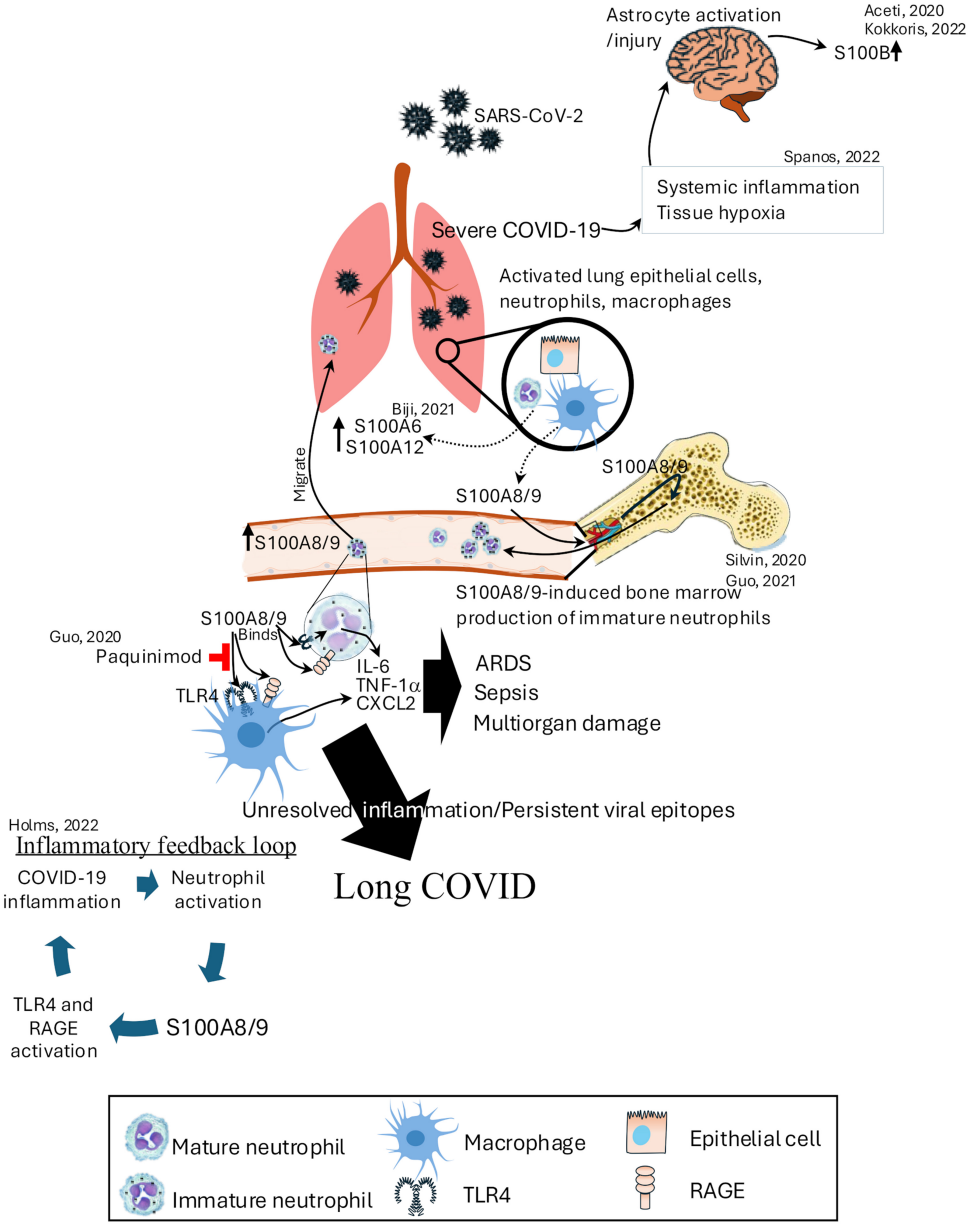
Role of S100 proteins in the pathogenesis of COVID-19 and long COVID. SARS-CoV-2 infection activates lung epithelial cells, neutrophils, and macrophages, leading to the release of S100A8/9, which binds to TLR4 and RAGE receptors. This interaction triggers an inflammatory feedback loop, promoting neutrophil activation and the production of immature neutrophils in the bone marrow. Elevated S100A8/9 levels contribute to severe COVID-19 by inducing ARDS, sepsis, and multiorgan damage through the release of IL-6, TNF-α, and CXCL2. On the other hand, severe COVID-19 increases the systemic inflammation and tissue hypoxia that leads to increased expression of S100B levels. Persistent viral epitopes and unresolved inflammation perpetuate long COVID, with S100A8/9 continuing to drive TLR4 and RAGE activation. ARDS, acute respiratory distress syndrome; CXCL2, chemokine (C-X-C Motif) ligand 2; TNF-α, tumor necrosis factor-alpha.

In the context of long COVID, sustained elevation of S100A8/A9 and inflammatory cytokines like IL-1β, IL-6, and TNFα indicate a chronic pro-inflammatory state, driven by a TLR4/RAGE feedback loop (178). This ongoing inflammation contributes to multi-organ symptoms such as fatigue, brain fog, and persistent inflammation, even after the virus is cleared (179). The continuous expression of proinflammatory cytokines is key to maintain long COVID symptoms (180). Targeting S100 proteins and their pathways offers a potential therapeutic strategy in this condition. Early treatments using inhibitors like ezrin peptides (181) and tocilizumab show promise in disrupting this inflammatory cycle (182). Additionally, inhibition of the binding of S100A8/A9 to TLR4 by paquinimod has shown it can reverse abnormal neutrophil activity and reduce mortality in coronavirus-infected mice (183). Additionally, long-term longitudinal studies have revealed specific perturbations in the immune system, including upregulated expression of S100A8/A9 and associated markers, even 6 months after acute SARS-CoV-2 infection (40). This persistent immune activation underscores the potential for S100 proteins to serve as both biomarkers and therapeutic targets in the management of COVID-19 and its long-term sequelae (Figure 5).

## S100 proteins as early biomarkers and therapeutic targets of graft rejection in lung transplantation

In the context of lung transplantation, elevated levels of S100 proteins such as S100A8, S100A9, and S100A12 can serve as early biomarkers of graft rejection or complications like graft-vs.-host disease. Higher plasma S100A8/A9 levels are associated with prolonged ischemic times and poorer outcomes post-lung transplantation. Treatment with an anti-S100A8/A9 antibody in bronchiolitis obliterans syndrome post-lung transplantation reduces myofibroblast infiltration and inflammation. Because of the damage-associated molecular patterns (DAMPs), they interact with receptors like TLR4 and RAGE, leading to the recruitment and activation of immune cells and the secretion of pro-inflammatory cytokines (184, 185). This inflammatory response can be indicative of transplant rejection or other immune-mediated events, making S100 proteins valuable for monitoring and managing post-transplant inflammation and immune responses in lung transplant patients.

## Clinical relevance and biomarker potential

S100 proteins mediates its effect through signaling pathways like RAGE and TLR4, influencing inflammatory mechanisms common to many lung diseases. Their functions vary by context, for example, S100A4 is involved in both tissue remodeling and metastasis, while S100A11 affects inflammation and chemotherapy resistance depending on the microenvironment. These insights suggest S100 proteins could serve as biomarkers for disease severity, prognosis, and therapeutic response; for instance, high levels of S100A8/A9 may indicate severe COVID-19 or pulmonary fibrosis, and S100A12 and S100A8/A9 can help monitor graft rejection in lung transplant patients. The main challenge lies in validating these proteins as reliable biomarkers and integrating them into clinical practice.

## Conclusion

In recent years, there has been significant progress in unraveling the roles of S100 proteins in pulmonary diseases, offering potential therapeutic avenues. Despite advancements in understanding S100 protein biology, gaps persist in comprehending the mechanism of many S100 proteins in the etiology of many diseases. Interestingly, the ongoing COVID-19 pandemic has brought to light the potential implication of S100 proteins in tissue damage, highlighting the imperative for further exploration in this field. Continued research on the intricate interactions and signaling mechanisms of S100 proteins is crucial for devising diagnostic biomarkers and innovative therapeutic targets to tackle lung diseases effectively. The ongoing research on S100 proteins may promise future development of tailored therapies in the domain of respiratory medicine.

### Points for clinical practice and future research

S100 proteins are emerging as promising biomarkers and therapeutic targets, with significant potential in lung diseases, such as elevated levels of S100A8/A9 (calprotectin) correlating with severe COVID-19 and cytokine storms, which suggests their utility as predictive markers. Monitoring these proteins may also help identify patients at risk for long COVID. Given their role in inflammation, airway remodeling, and tumor progression, S100 proteins are valuable for therapeutic development in pulmonary diseases. However, further research is needed to understand their molecular mechanisms in inflammation, protein-protein interaction, and synergy with other S100 proteins in disease progression and tumor metastasis, as well as their broader potential as cross-disease biomarkers, to enhance clinical applications.
